# A Stochastic Point Cloud Sampling Method for Multi-Template Protein Comparative Modeling

**DOI:** 10.1038/srep25687

**Published:** 2016-05-10

**Authors:** Jilong Li, Jianlin Cheng

**Affiliations:** 1 Department of Computer Science, University of Missouri, Columbia, MO 65211, USA; 2 Informatics Institute, University of Missouri, Columbia, MO 65211, USA

## Abstract

Generating tertiary structural models for a target protein from the known structure of its homologous template proteins and their pairwise sequence alignment is a key step in protein comparative modeling. Here, we developed a new stochastic point cloud sampling method, called MTMG, for multi-template protein model generation. The method first superposes the backbones of template structures, and the Cα atoms of the superposed templates form a point cloud for each position of a target protein, which are represented by a three-dimensional multivariate normal distribution. MTMG stochastically resamples the positions for Cα atoms of the residues whose positions are uncertain from the distribution, and accepts or rejects new position according to a simulated annealing protocol, which effectively removes atomic clashes commonly encountered in multi-template comparative modeling. We benchmarked MTMG on 1,033 sequence alignments generated for CASP9, CASP10 and CASP11 targets, respectively. Using multiple templates with MTMG improves the GDT-TS score and TM-score of structural models by 2.96–6.37% and 2.42–5.19% on the three datasets over using single templates. MTMG’s performance was comparable to Modeller in terms of GDT-TS score, TM-score, and GDT-HA score, while the average RMSD was improved by a new sampling approach. The MTMG software is freely available at: http://sysbio.rnet.missouri.edu/multicom_toolbox/mtmg.html.

The tertiary structure of a protein, which can be represented by the coordinates of its atoms, is important for understanding the function and activity of the protein^1^,^2^. Experimental techniques such as x-ray crystallography and nuclear magnetic resonance (NMR) can determine protein tertiary structure, but they are time-consuming and expensive, leading to a large gap between the number of known protein sequences (~100 million) and the number of known protein structures (~100,000). Therefore, computational protein structure modeling that provides a fast way of constructing an approximated structural model for a protein becomes increasingly popular and important^3^.

Computational protein modeling methods are usually classified into two categories: template-based modeling (TBM) that uses known protein structures as templates^3^,^4^,^5^,^6^,^7^,^8^,^9^,^10^,^11^,^12^,^13^ and template-free modeling (FM) that tries to build models from scratch without referring to any known structure^3^,^10^,^11^,^14^,^15^. Template-based modeling constructs protein structures by comparing a target sequence to template sequences in order to find homologous templates with known structures, and then transfer the template structures to the target protein through comparative modeling^3^. Using multiple templates, if available, generally improves the quality of models over using single template as demonstrated in the past Critical Assessments of Techniques for Protein Structure Prediction^16^,^17^,^18^, even though dealing with multiple templates that have some conflicting structural conformations is more difficult than handling a single template.

Generating protein 3D models for a target protein from its sequence alignment with template proteins and template structures is one of the most challenging steps in template-based modeling. Several methods were developed to address this problem, such as Modeller^19^,^20^, SWISS-MODEL^21^, ModSeg/ENCAD^22^, NEST^23^, etc. But all of these tools were initially developed more than a decade ago. SWISS-MODEL builds the core of a model by averaging the coordinates of backbone atoms in template structures, and uses the constraint space programming to construct the conformation of gaps – the region of a target protein not covered by any template^21^. ModSeg/ENCAD uses the segment match modeling to build models for a target protein^22^. NEST iteratively inserts/deletes one residue into/from template structure in order to build the whole model for target protein by minimizing the energy^23^. Modeller^19^,^20^ extracts spatial restraints from target-template alignments and template structures, and builds models for target protein by minimizing the restraint violations. Modeller, initially developed more than 20 years ago, is still the most widely used tool for generating structural models from target-template sequence alignments and template structures. The multiple-template threading method in Dr. Xu’s group uses a novel probabilistic-consistency algorithm to improve protein 3D modeling by accurately aligning a single protein sequence simultaneously to multiple templates^12^. The method can build protein models with better quality than single-template models even if the models are built from the best single template^12^. The probabilistic multi-template protein homology modeling method^13^ computes improved spatial restraints and calls Modeller to build 3D models. The method uses two-component Gaussian mixture distributions to combine density functions by multiplication compared to Modeller’s one-component densities. It also proposes new algorithms for computing template weights and selecting templates. Despite the importance of model generation, few new methods have been proposed to address some unsolved challenges associated with it.

One major challenge in comparative model generation is to integrate multiple templates to generate better models than single-template, which is particularly challenging when multiple templates suggest inconsistent or divergent conformations for the same region for target protein^24^. Another challenges including how to handle the noise in template conformations due to sequence divergence between target and template proteins and erroneous sequence alignments, and how to build conformations for gapped regions in a target protein that are not aligned with any template. Gaps may appear either in the middle of a target protein or at its terminals. Several popular methods such as SWISS-MODEL or Modeller either do not build conformations for long gaps or use a long extended chain to represent their conformations without trying to fold it.

In this study, we developed a new stochastic point cloud sampling method for Multi-Template Model Generation (MTMG) to address the challenges in generating structural models from multiple templates. Different than Modeller that extracts pairwise distance restraints for pairs of atoms or angular restraints of individual residues from templates, our method generate positional (x, y, z coordinate) restraints (point cloud) for each residue from templates centered on the weighted average structure of superimposed template structures. The point cloud is represented by a three-dimensional multivariate distribution, whose variance measure how uncertain the position of a residue is. The position of residue with low variance is generally fixed and that of residues with high variance is resampled from the distribution. A stochastic resampling move is rejected or accepted according to a simulated annealing protocol based on the RW protein energy function^25^ and the spatial distance restraints. The resampled model with the lowest energy is selected as the final model for the target protein.

We benchmarked MTMG on 1,033 sequence alignments generated for hundreds of targets used in the 9^th^, 10^th^ and 11^th^ Critical Assessment of Techniques for Protein Structure Prediction (CASP9, CASP10 and CASP11). MTMG of using multiple templates performed significantly better than using single template. Its performance is also comparable to the state-of-the-art model generation tool - Modeller - in terms of GDT-TS score (Global Distance Test Total Score)^26^, TM-score (Template Modeling score)^27^, and GDT-HA score (Global Distance Test High Accuracy score)^28^,^29^. In terms of RMSD (Root Mean Square Deviation), MTMG performs substantially better than Modeller by folding long gaps in target protein better.

## Results

In this section, we first briefly describe the sampling method of MTMG, and then present an evaluation of its performance from various perspectives.

### The sampling method of MTMG

Given a sequence alignment involving a target protein and one or multiple template proteins and the tertiary structures of the templates as input, MTMG first removes structurally inconsistent templates in order to decrease structural noise. The remaining templates are structurally superposed together, and the weighted average coordinates are calculated for each residue of the target. It then uses a *stochastic point cloud sampling* method to sample positions for the residues with uncertain conformations (i.e. unfixed residues) based on a three-dimensional multivariate normal distribution. The sampled positions iteratively replace the coordinates of the unfixed residues according to a simulated annealing protocol. A model with the lowest energy is selected as final prediction. The details of this modeling process are described in the Methods section.

### Benchmark datasets

**Figure 1:**
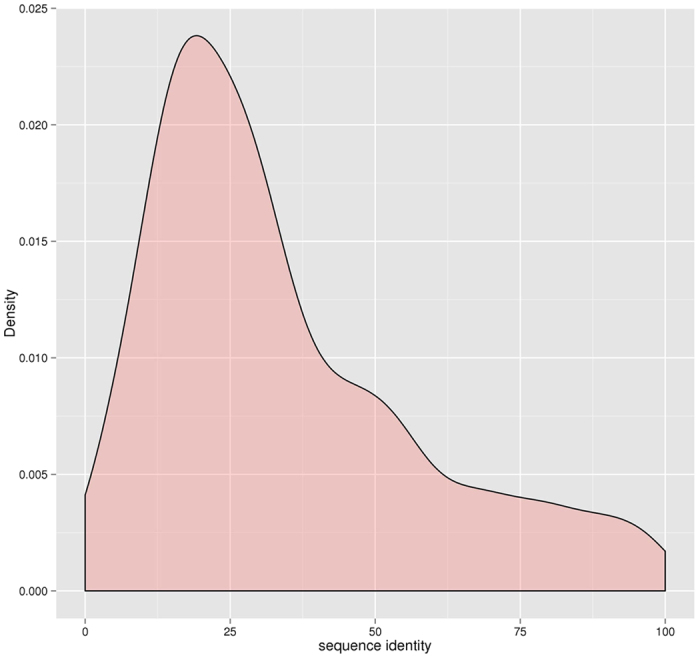
The distribution of sequence identity in the sequence alignments.

In order to rigorously evaluate our method, we benchmark MTMG on the three datasets, i.e. 104, 87, and 79 official targets in the 9^th^, 10^th^ and 11^th^ Critical Assessment of Techniques for Protein Structure Prediction (CASP9, CASP10 and CASP11), separately. In total these datasets have 1,033 target-template sequence alignments generated by different alignment tools used with our MULTICOM protein structure prediction server^30^,^31^,^32^,^33^,^34^,^35^. CASP9, 10, 11 datasets have 398, 313, and 322 target-template sequence alignments, respectively, which were generated by HHSearch (versions 1.2 and 1.5)^36^, HMMer^37^, and CSI-BLAST^38^ separately under same condition. HHSearch^36^ is a profile-profile alignment tool. HMMer^37^ is a profile-sequence alignment tool based on profile hidden Markov models. CSI-BLAST^38^ is a tool for iterative search of homologues with position-specific scoring matrices. The pairwise alignments between a target and each of multiple templates produced by these tools were combined into multiple sequence alignments^30^,^31^,^32^,^33^,^34^ in order to use multiple templates if exist. At the end, 299 (75.13%) CASP9 alignments, 243 (77.64%) CASP10 alignments, and 259 (80.43%) CASP11 alignments contain multiple templates. Figure 1 shows the distribution of sequence identity in the sequence alignments. Most top template sequences have sequence identity of ~20% with the target sequence, and the average sequence identity is 34.23%. The datasets are sufficiently large and contain diverse types of sequence alignments and targets, making them good datasets to objectively benchmark our method. The sequence alignments, template structures, and predicted models in this study are available at http://sysbio.rnet.missouri.edu/multicom_toolbox/mtmg.html.

We ran MTMG and a state-of-the art comparative modeling tool - Modeller on the three CASP datasets to predict 3D structures for the CASP targets in order to compare their performance. The default approach in Modeller was used to generate 25 structural models for each sequence alignment. The model with lowest DOPE score calculated by Modeller was used for benchmark. MTMG was also run on each single template to generate single template-based models in order to study if and how multiple templates may improve modeling performance. The predicted models were superposed with true structures to calculate GDT-TS score, TM-score, GDT-HA score, and RMSD using the TM-score program^27^.

### Multiple templates versus single template

We compared the models predicted by MTMG on multiple templates and on the first single template selected by each alignment tool. Table 1 reports the average GDT-TS score, TM-score, GDT-HA score, and RMSD of the models based on the first single template and on multiple templates on CASP9, CASP10, and CASP11 targets. The results show that using multiple templates improved GDT-TS score (or TM-score) by 6.37%, 3.65%, and 2.96% (or 5.19%, 3.15%, and 2.42%) over using first single template on the three data sets separately. The average RMSD was also obviously improved by using multiple templates.

Table 2 reports the p-values of t-test^39^ on GDT-TS score and TM-score for comparison between single template and multiple templates on CASP9, CASP10, and CASP11 targets. All the p-values are <0.05, indicating that using multiple templates can significantly improve the global quality of the predicted models in terms of GDT-TS score and TM-score over using single template, which is the generally the alignment with the lowest e-value calculated by a sequence alignment tool.

**Figure 2:**
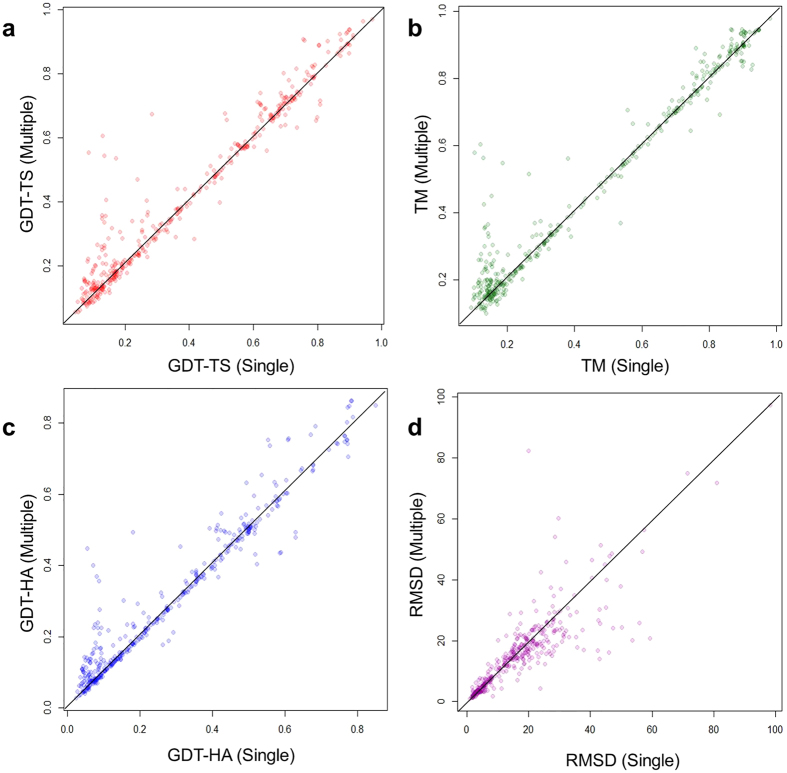
The improvements or losses of GDT-TS score, TM-score, GDT-HA score and RMSD of the models predicted by MTMG using the first single templates and multiple templates on individual CASP11 targets. The scores of multi-template models are plotted against single-template models. X-axis represents the scores of single-template models and Y-axis represents the scores of multi-template models.

We investigated improvements or losses of GDT-TS score, TM-score, GDT-HA score, and RMSD on individual targets. Figure 2 shows scatter plots of GDT-TS scores, TM-scores, GDT-HA scores, and RMSDs between the single-template models and the multiple-template models on CASP11 targets. From the figure, using multiple templates improves the predicted models on all the scores. Since the first single template is considered the most relevant (or significant) template selected by each sequence alignment tool, the results suggest using multiple template consistently improve the quality of comparative modeling over using the top one single template selected by an alignment tool. The results are consistent with the previous studies^16^,^17^,^18^.

**Figure 3:**
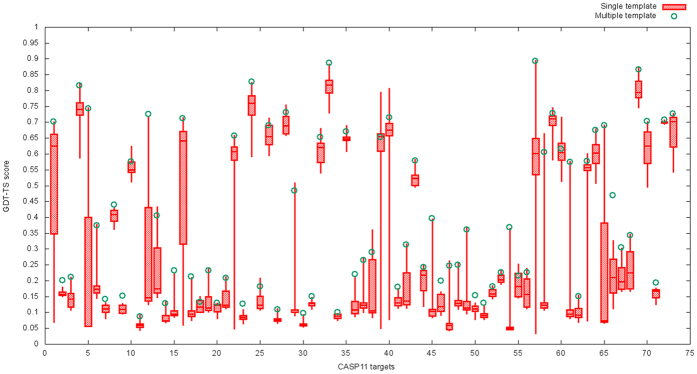
The boxplot of GDT-TS scores of the models predicted by MTMG for each of 73 CASP11 domains using each single template and multiple templates. The box plot denotes the maximum, 75% quartile, mean, 25% quartile, and minimum score of the models constructed from each single template for a target. The small green circle denotes the score of the model constructed from multiple templates.

We chose 51 CASP11 targets (73 domains), which were covered by at least three templates, in order to compare the multi-template models with all possible single-template models, i.e., the models generated by every single template in the alignments. These targets have between 3 and 41 templates. The minimum, maximum, median, quartile at 25 percentile, and quartile at 75 percentile of GDT-TS score of single-template models were calculated. Figure 3 shows the GDT-TS score of the predicted models on 73 CASP11 domains using single templates and multiple templates. The multi-template models have higher GDT-TS score than median of the GDT-TS scores of the single template models on 72 domains. The average improvement of GDT-TS score is 0.1188. The results illustrate that using multiple templates can improve the quality of the predicted models substantially. Furthermore, the multi-template models have higher GDT-TS score than the best models built from the best possible single template on 30 domains. The average difference of GDT-TS score between multi-template models and best-template models is almost zero (i.e., −0.0065) and the most significant improvement is 0.1448, indicating that using multiple templates yields the similar performance with using the best single template. Since it is impossible to select the best template for each target without knowing the true structures of the target most time, using multiple templates is the practical way to achieve the best potentials within template structures.

### MTMG versus Modeller

We compared MTMG with Modeler on CASP9, CASP10, and CASP11 targets. Table 3 reports the average GDT-TS score, TM-score, GDT-HA score, and RMSD of the models predicted by MTMG and Modeller on CASP9, CASP10, and CASP11 targets, respectively. The average GDT-TS score and TM-score on CASP11 targets and the average TM-score on CASP10 targets of MTMG are slightly higher than that of Modeller, while the average GDT-TS score on CASP10 targets and the average GDT-TS score and TM-score on CASP9 targets of MTMG were slightly lower than those of Modeller. One the super dataset that combine all CASP9, CASP10, and CASP11 datasets together, the average GDT-TS score and TM-score of MTMG is 0.5121, 0.5570, which is similar to 0.5136 and 0.5543 of Modeller. Overall, the performance of MTMG is comparable to Modeller in terms of GDT-TS score and TM-score. However, in terms of RMSD, MTMG performed better than Modeller. According to Table 3, the average RMSD of MTMG was 5.96 Å, 13.29 Å, and 3.91 Å lower than that of Modeller on the three datasets, respectively. The reason of the improvement is that MTMG models long gaps in target proteins that are not covered by templates better than Modeller. MTMG and Modeller use similar approaches to model the unaligned regions which no template covers. Both of them loop the unaligned regions out into space. MTMG uses the spatial restraints to sample conformations for long gaps and chooses the angles at random. Modeller chooses always same angle and usually generates an unfolded stick for a long gap. Modeller’s method directly shows where no alignment information exists. In the contrast, our method tries to make a reasonably folded conformation for the long gap. This may be a reason that the RMSD of the unaligned regions in MTMG models is averagely lower than that in Modeller models.

**Figure 4:**
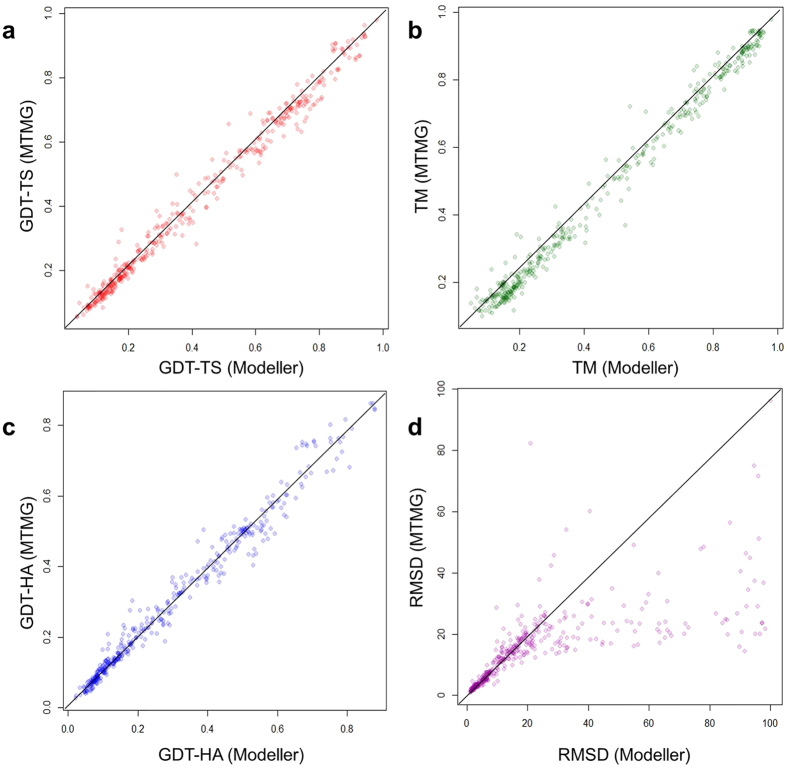
The scatter plot of GDT-TS scores, TM-scores, GDT-HA scores and RMSDs of the models predicted by MTMG against those of Modeller on CASP11 targets. The scores of Modeller models are plotted against MTMG models. X-axis represents the scores of Modeller models and Y-axis represents the scores of MTMG models.

We compared the improvements or losses of GDT-TS score, TM-score, GDT-HA score, and RMSD of MTMG and Modeller on the individual targets of the three datasets. Figure 4 shows the scatter plots of GDT-TS score, TM-score, GDT-HA score, and RMSD between the MTMG models and the Modeller models on CASP11 targets. The figure shows that the performance of MTMG is comparable to that of Modeller in terms of GDT-TS score, TM-score and GDT-HA score, while RMSD of MTMG is significantly lower than that of Modeller.

We also compared our method with the probabilistic multi-template protein homology modeling method^13^. The probabilistic multi-template method relies on HHSearch’s output (.hhr) to generate spatial restraints and calls Modeller to build 3D models. In the contrast, our method is an independent method and doesn’t rely on outputs of any specific tools and any model generation tools. Table 4 shows average GDT-TS score, TM-score, GDT-HA score, and RMSD of the models predicted by MTMG and the probabilistic multi-template method based on HHSearch’s outputs on CASP9, CASP10, and CASP11 targets. From the table, the performance of the probabilistic multi-template method is better than that of our method on GDT-TS score, TM-score, and GDT-HA score. However, our method improved RMSD over the method. We didn’t do more comparisons between our method and the probabilistic multi-template method because they accept different kinds of input files.

**Figure 5:**
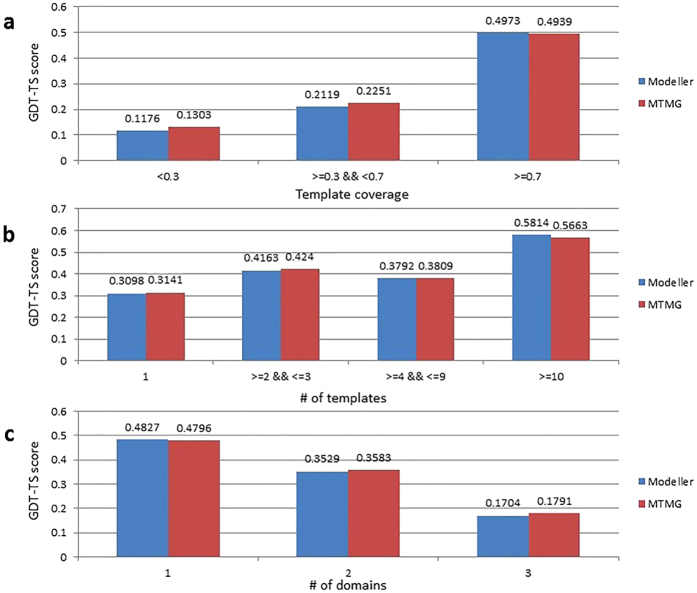
Comparison of GDT-TS score between the MTMG models and the Modeller models from three aspects on CASP11 targets. **(a)** MTMG performed better than Modeller on targets with <0.7 template coverage. **(b)** MTMG performs better than Modeller on targets covered by <10 templates. **(c)** MTMG performs better than Modeller on targets containing multiple domains.

We compared MTMG with Modeller on CASP11 targets with different template coverage in order to elucidate how they performed differently. Figure 5a shows the comparison of GDT-TS score between the MTMG models and the Modeller models with different template coverage. X-axis represents template coverage, and y-axis represents the average GDT-TS score. According to the results, MTMG performed better than Modeller on targets with <0.7 (e.g. 70%) template coverage, but slightly worse than Modeller on targets with > = 0.7 template coverage. The improvement on targets with lower template coverage by MTMG was partially due to its capability of sampling the conformation of long gaps.

We checked how the number of templates might affect the performance of MTMG and Modeller. Figure 5b compares the GDT-TS score between the MTMG models and the Modeller models constructed from different numbers of templates. X-axis represents the number of templates, and y-axis represents the average GDT-TS score. The results show that, MTMG performed better than Modeller on targets covered by <10 templates, while it performed worse than Modeller on targets covered by >10 templates.

We further compared the performance of MTMG with Modeller on CASP11 targets containing different numbers of domains. Figure 5c reports the GDT-TS score of the MTMG models and the Modeller models for targets with different numbers of domains. X-axis represents the number of domains, and y-axis represents the average GDT-TS score. MTMG performed better than Modeller on targets containing multiple domains. The results suggest that the domain division and combination protocol used by MTMG can improve the quality of modeling multi-domain proteins.

We classified CASP9, 10 and 11 targets into four SCOP protein categories: all-alpha, all-beta, alpha/beta, and alpha+beta. Table 5 shows average GDT-TS score of MTMG and Modeller models on four protein categories on CASP targets. From the table, the class of the proteins doesn’t have an obvious impact on the quality and improvement of MTMG models.

We divided CASP9, 10 and 11 targets into easy, medium, and hard targets according to CASP official classification. The average GDT-TS score and RMSD were calculated for different kinds of targets as shown in Table 6. From the table, RMSD was improved by our method on any kinds of targets. GDT-TS score was improved by our method on medium and hard targets. For easy targets, the performance of our method on GDT-TS score was a little bit worse than that of Modeller.

**Figure 6:**
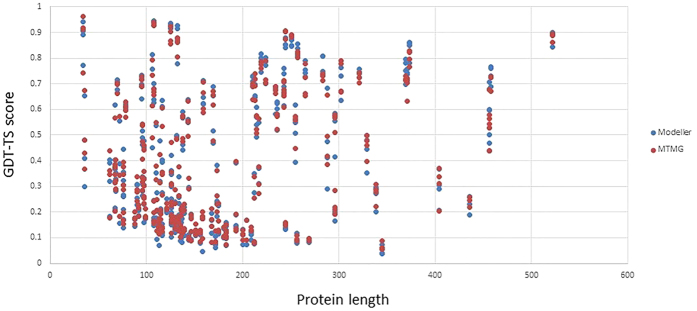
The GDT-TS scores of MTMG and Modeller models on different protein lengths. Red points donate GDT-TS scores of MTMG models, and blue points donate GDT-TS scores of Modeller models.

We also analyzed GDT-TS score of MTMG and Modeller models on different protein lengths for CASP9, 10, and 11 targets. The analysis results are shown on Fig. 6. Red points donate GDT-TS scores of MTMG models, and blue points donate GDT-TS scores of Modeller models. The figure shows that GDT-TS scores of MTMG and Modeller models are mostly similar on different protein lengths. It implies that the length of the proteins doesn’t have a critical impact on our method.

In addition to comparing MTMG and Modeller in terms of global backbone quality scores such as GDT-TS score, TM-score, and RMSD, we compare them in terms of MolProbity score that measures the “realistic” level of models. MolProbity is a knowledge based metrics that evaluates the physical reasonableness of molecular models^40^. The models generated by our method have higher average MolProbity scores (i.e., worse local quality) than Modeller. For example, the average MolProbity score of the MTMG models is 3.51, which is higher than 3.02 of the Modeller models on CASP11 targets. The problem may be caused by the way used by MTMG to convert the reconstructed Cα trace into a full backbone. A solution to improve the MolProbity score is to use ModRefiner^41^ to generate main-chain and side-chain atoms from Cα trace instead of using Pulchra. According to our experiment on CASP11 targets, with a very slight decrease in GDT-TS score by 0.008, the average MolProbity score of MTMG models could be improved to 2.90 by ModRefiner, which is better than the average MolProbity score of the Modeller models. Therefore, if necessary, users can run ModRefiner on MTMG’s models to generate full-atom models with good MolProbity scores.

### The impact of simulated annealing protocol

**Figure 7:**
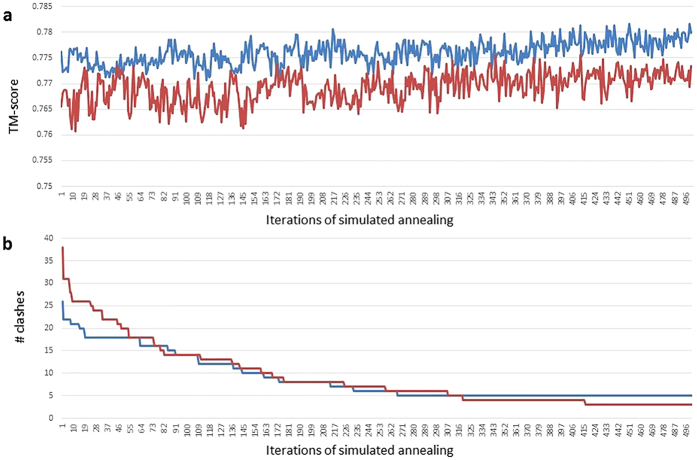
Changes of TM-score (**a**) and the number of atom clashes (**b**) of the models for two CASP11 targets during the simulated annealing. TM-score stochastically went up and down with an overall upward trend during simulated annealing. Even though the final model was not the best one, but it was close to the best one and better than the initial model. Moreover, the number of clashes rather consistently decreased during simulated annealing.

MTMG uses simulated annealing to iteratively generate new models with sampled points. We investigate how it can improve the quality of models. Figure 7 shows the changes of TM-score (a) and the number of clashes (b) of two CASP11 targets with respect to iterations during simulated annealing. The plots show that TM-score stochastically went up and down with an overall upward trend during simulated annealing. Even though the final model was not the best one, but it was close to the best one and better than the initial model. Moreover, the number of clashes rather consistently decreased during simulated annealing.

### Several case studies

**Figure 8:**
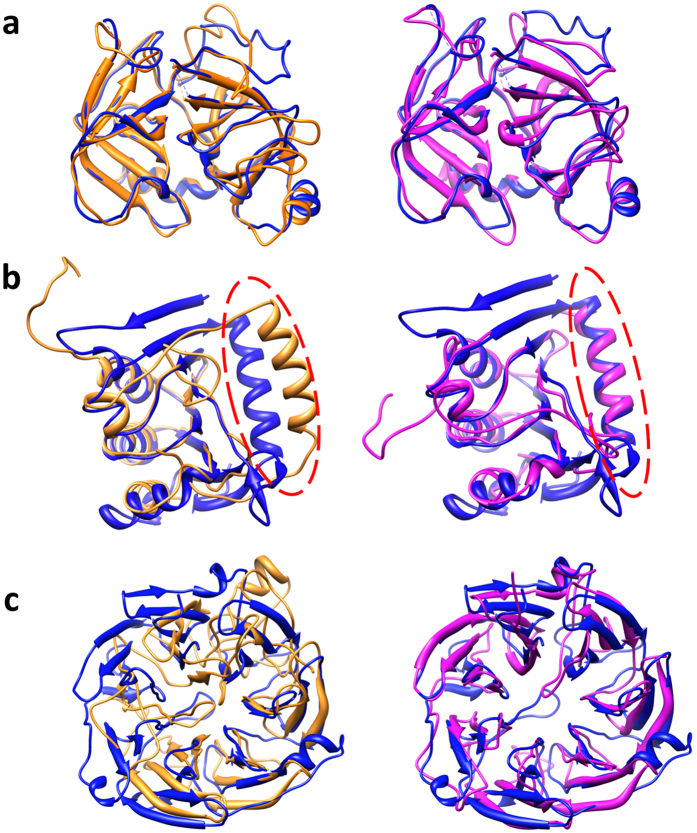
Three examples illustrating (**a**) the successful template weighting and combination, (**b**) the successful template superposition, and (**c**) the successful domain division and combination of our method. The models predicted by Modeller (gold) and MTMG (purple) were superposed with the native structure (blue).

We studied several cases on which MTMG performed better than Modeller to demonstrate how MTMG improved the quality of modeling. Figure 8a shows the structural superposition between the native structure (blue) of target T0841 and the models predicted by Modeller (gold) and MTMG (purple). The GDT-TS scores of the Modeller model and the MTMG model were 0.8524 and 0.9058 respectively. The target protein has only one domain, covered by 78 significant templates with e-values of alignment equal to 0. The structural inconsistency within 78 templates may have reduced the quality of Modeller models. After removing inconsistent templates by structural superposition, MTMG chose 10 templates to construct models leading to better quality.

Figure 8b illustrates the structural superposition between the native structure (blue) of target T0847 and the models predicted by Modeller (gold) and MTMG (purple). The GDT-TS scores of the Modeller model and the MTMG model were 0.5258 and 0.6553, respectively. MTMG improved GDT-TS score by 0.1295. The region (residues 148–168) of the MTMG model circled by red is superposed with the native structure much better than that of the Modeller model circled by red. The target protein is covered by two templates 1BYRA and 4GGKA, which cover 10 common residues (residues 129–138) of the target. The circled region is covered by 4GGKA. MTMG superposed 4GGKA against 1BYRA in order to align them to the correct location. The result shows that the MTMG’s process of template superposition successfully aligns the templates together to improve the quality of modeling.

Figure 8c compares the native structure of domain T0845-D2 (blue) with the Modeller model (gold) and the MTMG model (purple). The CASP11 target T0845 has two domains: T0845-D1 (residues 23–119) and T0845-D2 (residues 120–448). In the sequence alignment, the target protein is covered by two templates 3TC9A and 3DSMA. 3TC9A covers residues 34–112 and 3DSMA covers residues 122–448. But the two templates do not cover any common residues. So, MTMG divided the target protein into two domains: D1 (residues 1–119) and D2 (residues 120–448). The two domains were modeled separately, and the predicted models of the two domains were combined into a full-length model using the moving and rotating algorithm. The GDT-TS scores of the Modeller model and the MTMG model were 0.3574 and 0.4985, respectively. MTMG improved GDT-TS score by 0.1411 on domain T0845-D2. For domain T0845-D1, the GDT-TS scores of the Modeller model and the MTMG model were 0.3262 and 0.3582 separately. MTMG improved GDT-TS score by 0.032. In this case, MTMG’s domain division and combination protocol improved the quality of modeling.

### Running time

**Figure 9:**
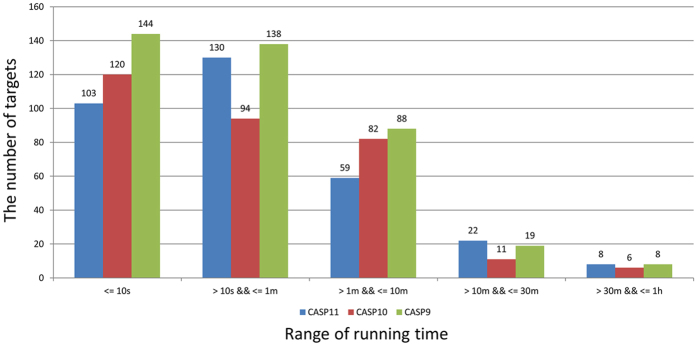
The number of targets in different ranges of running time on CASP9, CASP10, and CASP11 targets. 92.83% of targets were modeled by MTMG within 10 minutes, and all the targets were modeled in an hour in the experiment.

We investigated the running time of our method in the experiment. MTMG was run on single CPU with the x86_64 Red Hat Linux system. Figure 9 shows the number of targets in the different ranges of running time on CASP9, CASP10, and CASP11 targets. The running time of 92.83% of targets is < = 10 minutes, which is reasonably fast. The minimum running time on three datasets is 1 second. The average running time on CASP9, CASP10, and CASP11 targets is 2′22″, 2′28″, and 3′ separately. The maximum running time on these datasets is 44′38″, 56′58″, and 56′44″ separately. The speed is related to template similarity, target length, the number of templates, and the number of gaps. The minimum running time occurred on targets with single template or structurally very similar templates, and good template coverage. The maximum running time occurred on long targets with many unfixed residues and/or gaps. We also tested the speed of Modeller on same condition. Modeller usually spent a few seconds to several minutes to build a structural model. Although our method is fast, it is a little bit slower than Modeller on average.

## Discussion

In this study, we designed and implemented a new stochastic point cloud sampling method for multi-template protein model generation (MTMG) in comparative modeling. The stochastic sampling and simulated annealing protocol in MTMG has the capability to improve the global quality and reduce atom clashes in models. Some new techniques are developed to improve modeling, including the template superposition and weighting for removing structural inconsistency and considering the relevance of templates, domain division and combination for integrating overlapped templates, and moving and rotation algorithm for loop (gap) modeling. Our extensive experiment on three CASP datasets clearly demonstrates that using multiple templates significantly improves the performance of comparative modeling over using first/average single templates, and the performance of using multiple templates is comparable to the idea case of using the best possible templates available. On the same benchmark, the performance of MTMG is comparable to that of a state-of-the-art method – Modeller. The difference in the performance of MTMG and Modeller is related to the template coverage, the number of templates, and the number of domains of a target protein. Overall, MTMG is a new, complementary, and useful addition to the important, yet under-developed tool set for protein comparative modeling.

## Methods

### The stochastic point cloud sampling method (SPC) for sampling conformations from multiple templates

**Figure 10:**
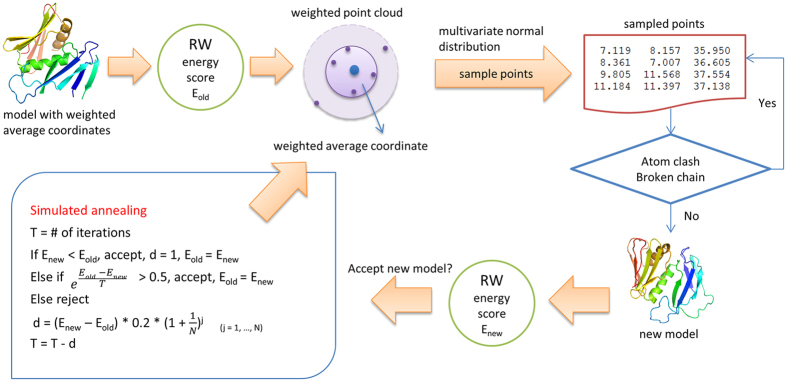
The workflow of the stochastic point cloud method for sampling conformations. Starting from an initial model comprised of the weighted average coordinates of template structures, its RW energy is calculated as E_old_, weighted point clouds are constructed for unfixed residues whose conformations are uncertain. New positions are sampled for unfixed residues from the multivariate normal distribution representing the point clouds, the positions with few or no atom clashes or broken chain are accepted to generate a new model. The new model is accepted based on the difference between its energy E_new_ and the old energy E_old_ according to a simulated annealing protocol, and the accepted model is used as the initial model for the next round of modeling, which is repeated until reaching a fixed number of iterations.

Figure 10 illustrates the workflow of the stochastic point cloud (SPC) sampling method for sampling conformations for a target protein covered by multiple templates. For a target protein covered only by a single template, the backbone of the template structure is copied directly to the predicted model without invoking the point cloud method. Otherwise, it works as follows.

The SPC method extracts a set of coordinates (*x*, *y*, *z* coordinates) of Cα atoms for residues in a target protein from the template structures according to target-template alignments if exists. For a Cα atom, it calculates the weighted average coordinates (i.e. the central coordinates) of the Cα atoms in multiple templates, the distance between the position of a Cα atom in each template and the weighted average coordinates, and the weighted average distance between Cα atoms in multiple templates. The set of coordinates of Cα extracted from the template structures define a weighted point cloud centered at the weighted average coordinates for each residue. The weighted average coordinates are calculated using equation (1):


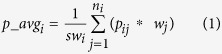


where *p_avg*_*i*_ is the weighted average coordinates of the i^th^ residue, *sw*_*i*_ is sum of weights of templates covering the i^th^ residue, *n*_*i*_ is the number of templates covering the i^th^ residue, *p*_*ij*_ is coordinates (x_ij_, y_ij_, z_ij_) of Cα of the i^th^ residue in the j^th^ template, and *w*_*j*_ is the weight of the j^th^ template.

The weighted average distance is calculated using equation (2):


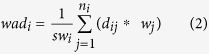


where *wad*_*i*_ is the weighted average distance of the i^th^ residue, *sw*_*i*_ is sum of weights of templates covering the i^th^ residue, *n*_*i*_ is the number of templates covering the i^th^ residue, *d*_*ij*_ is the distance between *p_avg*_*i*_ (weighted average coordinates) and *point*_*ij*_, and *w*_*j*_ is the weight of the j^th^ template.

The weighted average coordinates of Cα atoms are used as the initial model to be optimized. A global energy score for the initial model is calculated by the RW potential function^25^ and is denoted as E_old_. RW is a pairwise distance-dependent, atomic statistical potential function^25^.

For a residue, if its weighted average distance is >0.5 Å, the coordinates in the point cloud are considered significantly varied. Such a residue is called an unfixed residue whose conformation is largely uncertain or different in multiple templates and needs to be resampled. SPC samples the positions for unfixed residues using a three-dimensional multivariate normal distribution in order to find a better position to replace the old one. The probability density function of the *d*-dimensional multivariate normal distribution is given by equation (3):


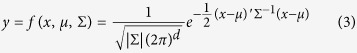


where *μ* represents the 1-by-*d* mean vector (i.e., weighted average coordinates), Σ represents the *d*-by-*d* covariance matrix (i.e., weighted average distance), and *x* represents a 1-by-*d* random variable (sampled point or position). Σ is calculated using equation (4):


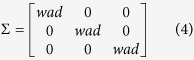


where *wad* represents weighted average distance. The diagonal elements of Σ contain the variances for each variable (i.e. each coordinate), which is set approximately by the weighted average distance by default. The off-diagonal elements of Σ contain the covariance between variables, which are set to 0 assuming there is no covariance between variables.

The multivariate normal distribution provides an effective, density-based clustering method for sampling points from a 3D space defined by the point cloud of the Cα atom of a residue^42^,^43^.

SPC uses the function *mvrnorm* in R package ‘MASS’^44^. The function is called to sample 100 points for each unfixed residue in each iteration.

**Figure 11:**
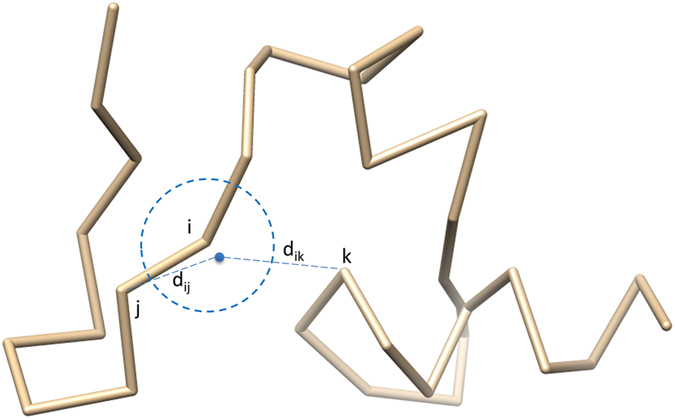
Checking the validity of sampled points. The Euclidean distance of the backbone atom Cα is calculated between the sampled point of the i^th^ residue and each of other residues. The sampled point is accepted if it satisfies the spatial restraints without broken chains (i.e. too far away from adjacent atoms: d_ij_ > 4.5 Å) and atom clashes (too close to other atoms: d_ik_ < 3.5 Å).

The sampled points are evaluated before being accepted or rejected (see Fig. 11). One sampled point is rejected if it causes a broken chain (the distance between two adjacent Cα atoms >4.5 Å) or atom-atom clashes (the distance between two Cα atoms <3.5 Å), otherwise is accepted. If a sampled point is accepted or all the sampled points are rejected within 100 tries, SPC will move to the next unfixed residue to sample its positions until the positions of all the unfixed residues are resampled. The accepted sampled points replace the current coordinates of the respective unfixed residues in the current model to generate a new model. A global energy score is calculated by RW^25^ for the new model as E_new_.

After a new model is sampled, SPC uses simulated annealing to decide whether the new model is accepted (or kept). Simulated annealing is a stochastic optimization technique to minimize or maximize an objective function^45^,^46^, i.e. to find the optimal model with minimum energy. The initial temperature is the number of iterations, which equals to 1,000 divided by the number of the unfixed residues, and is constrained in between 20 and 100 by default. If a number between 1 and 500 is given while running MTMG, the number of iterations equals to the number of unfixed residues multiplying the given number. The temperature decreases from iteration by iteration. If E_new_ is less than E_old_, the new model is accepted, E_old_ is set to E_new,_ and the temperature (T) decreases by 1. If E_new_ is greater than or equals to E_old_, a probability of accepting the new model is calculated as e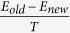. If the probability is >0.5, the new model is accepted, and otherwise it is rejected. And the temperature decreases by d, which is calculated using equation (5):


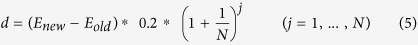


where N is the number of iterations. *E*_*new*_ − *E*_*old*_ makes that *d* is a positive number because *E*_*new*_ is larger than *E*_*old*_. We considered 5 as a basic difference between *E*_*new*_ and *E*_*old*_, so we multiplied it with 0.2 in order to make it around 1. 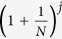 is getting bigger during iterations, which is consistent with simulation annealing.

The sampling process stops when the maximum number of iterations is reached or temperature drops to 0 or below. The last accepted model by the end of sampling is the final model predicted for a target.

**Figure 12:**
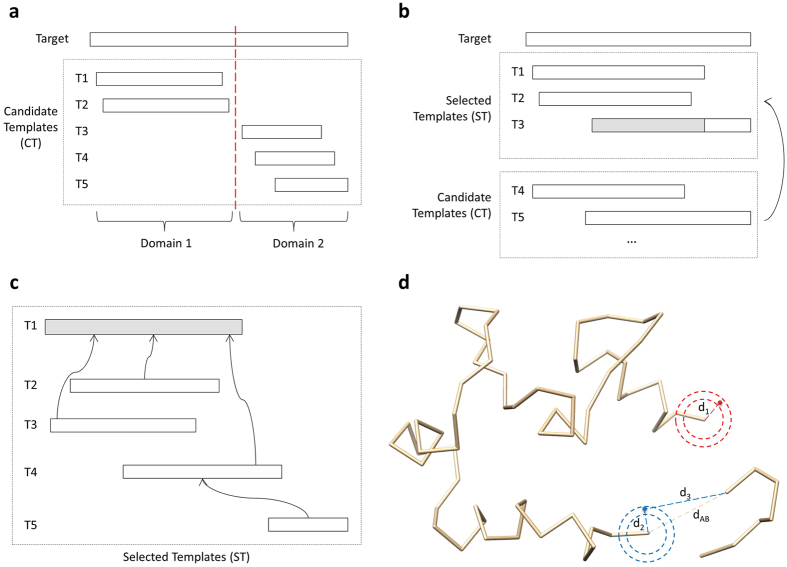
Domain division. (**a**) A target protein covered (aligned with) five templates is divided into two domains because the two regions do not share any common templates. (**b**) **Template combination.** The template T1 with the highest template weight is selected first. T2 is selected because the TM-score between T1 and T2 is >0.7. T3 is chosen because it covers at least 10 continuous uncovered target residues. **(c) Template superposition.** T1 is the center template. T2, T3, and T4 are superposed with T1 because they share common residues with T1. T5 does not share common residues with T1, so it is superposed with T4. **(d) Sampling points for gaps.** The radius of the outside circle is 4.5 Å, and the radius of the inner circle is 3.5 Å. The sampling algorithm randomly samples point between the two circles. In the region circled by red, the gap is at the N-terminal. The distance d_1_ between an accepted sampled point and the first covered residue is between 3.5 Å and 4.5 Å. In the region circled by blue, the three-residue gap is in the middle, and the distance between the two ends of the gap (d_AB_) is 8.2 Å. The distance d_2_ between an accepted sampled point and the last covered residue before the gap is between 3.5 Å and 4.5 Å. The distance d_3_ between an accepted sampled point and the first covered residue after the gap is between 4.1 Å and 11.4 Å.

The modeling method discussed above can be used directly to build models in most cases when a target is globally covered by at least one template or by multiple overlapped templates. For a target whose regions are only covered locally by different templates, we added one step of domain division and combination to join the conformations of regions covered by different templates into a full-length model. Figure 12a shows a target whose two domains are covered by five templates without overlapped linkers to join them together. The left region (domain 1) of the target is covered by templates T1 and T2, and the right region (domain 2) is covered by T3, T4, and T5. But the two regions are not covered by any common template. In this case, we divide the target protein into two domains and model them separately using the SPC method discussed above.

After the two domains are modeled, we use a moving rotation algorithm to combine the models of domains into a full-length model by iteratively combining models of two adjacent domains. The algorithm has the following six steps: (1) getting the coordinates of the last residue (A) in the first domain D1 and the coordinates of the first residue (B) in the second domain D2, and calculating the distance between A and B as d_AB_; (2) if d_AB_ is too small or too big for two adjacent residues (d_AB_ < 3.5 Å or d_AB_ > 4.5 Å), moving (translating) D2 to a new location so that d_AB_ is between 3.5 Å and 4.5 Å; (3) if there are less than 15 atom clashes between D1 and D2, no severe clashes and no broken chains, the algorithm exits, and otherwise continue to the following steps; (4) sampling points (positions) around A in order to find a point C so that the distance between A and C (d_AC_) is between 3.5 Å and 4.5 Å and there are no atom clashes between D1 and C; (5) moving (translate) D2 so that B and C are in the same position; and (6) iteratively rotating D2 in order to find its orientation where the combined model has no/least atom clashes. The rotation is implemented by equation (6):


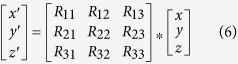


Given a unit vector u = (*u*_*x*_, *u*_*y*_, *u*_*z*_), where *u*_*x*_^2^ + *u*_*y*_^2^ + *u*_*z*_^2^ = 1, the matrix (R) used for rotation by an angle *θ* around an axis in the direction of u is calculated using equation (7)^47^:


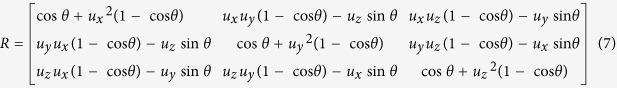


Different *u*_*x*_, *u*_*y*_, and *u*_*z*_ were drawn from the range of [−1, 1] and they changed by 0.1 for each iteration.

### Template weighting and combination

Before using the structures of the templates in model sampling as described above, all the templates are preprocessed in order to make them structurally comparable and consistent as follows. Residues in templates that do not cover the target protein are removed from the sequence alignment and template structures. The remaining residues and atoms are re-indexed according to their sequence alignments with the target protein.

The quality of the model constructed for the target protein depends on the quality and relevance of selected templates, such as sequence similarity between the target and templates and template coverage. Using multiple complementary templates can often reduce modeling variance and increase template coverage leading to better models, but low-quality templates may decrease the quality of models. Therefore, we assign a weight to a template to control its impact on calculating the average coordinates or the variance of point cloud for a residue. The weight of a template is the sum of five terms: average TM-score, template coverage, sequence identity, sequence similarity, and E ^− e-value^, which are described as follows: (1) *Average TM-score*. Each template is aligned with other templates by the TM-score program^27^ and a pairwise TM-score between 0 and 1 is calculated for each pair of templates. The average TM-score^26^ is calculated for each template. (2) *Template coverage*. It is the ratio between the number of residues covered by the template and the length of the target. (3) *Sequence identity*. It is the ratio between the number of identical residues between the target and the template in target-template alignment and the total number of target residues covered by the template. (4) *Sequence similarity*. The similarity score is calculated for each pair of residues in the target-template alignment using BLOSUM62 Matrix^48^. If the score is <0, score is set to e^score^; otherwise score is set to score plus 1. The sequence similarity for the template is calculated using equation (8):


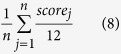


where *n* is the total number of target residues covered by the template (template coverage length), and *score*_*j*_ is the similarity score of the j^th^ residue (1 < = j < = n). *Score*_*j*_ is divided by 12 in order to scale it to the range [0, 1]. (5) *E*^*− e-value*^. An e-value measuring the significance of an alignment score is extracted from the sequence alignment for each target-template alignment. If an alignment does not provide e-value, an identical e-value (i.e. 0) is assigned to all the templates. The *E*^*− e-value*^ ranges between 0 and 1. Lower e-value (i.e. a more significant alignment score) leads to a larger *E*^*− e-value*^.

After the weights for all the templates are calculated, the template with the highest weight is first selected. For each of other candidate templates, if it covers at least 10 continuous target residues that are not covered by any of the selected templates, or if its pairwise TM-score with the template with the highest weight is >0.7, it is chosen. This step is repeated until all the candidate templates have been checked. For example, in Fig. 12b, T1 is the template with highest template weight, which is selected first. T2 is selected because the TM-score between T1 and T2 is >0.7. T3 is chosen because it covers at least 10 continuous uncovered target residues. However, the left region (gray) of T3 may be removed after template superposition if the TM-score between it and any selected template is <0.7. This process is to filter structurally inconsistent coordinates before averaging them in order to reduce structural noise.

The selected templates are then superposed by the TM-score program^27^ in order to make them structurally consistent and to make their averaged coordinates reasonable as follows. We used TM-score because we filtered unaligned residues from the template structures and the residue names in templates were replaced with those of the target protein. So, pre-handled templates were supposed to be generated from the same protein sequence. The template with the highest weight is selected as the center template. All the other templates are superposed with the center template if they share common residues with the center template. If a template does not share common residues with the center template, it is superposed with an already superposed template that shares most common residues with it. Figure 12c illustrates the template superposition protocol using five templates. T1 is the center template. T2, T3, and T4 are superposed with T1 because they share common residues with T1. T5 does not share common residues with T1, so it is superposed with T4. The superposed template structures only contain the x, y, z coordinates of Cα atoms. The superposed template structures are used to generate the average coordinates and the point clouds of the target residues.

### Modeling gaps

The residues in a target that are not covered by any selected template are gaps (e.g. loops). Our method models the conformations of gaps by iteratively sampling points based on spatial distance restraints. The restraints include: (1) the distance between any pair of Cα atoms should be >3.5 Å and (2) the distance between two adjacent Cα atoms should be <4.5 Å. Terminal gaps or internal gaps are handled differently as follows.

(1) Gaps at N terminal or C terminal

*A* = *the first covered target residue adjacent to the gap at the N-terminal or the last covered target residue adjacent to the gap at C-terminal;*

*While there is gap*

*  Sample points around A;*

*  Find point B where there is no atom clash and the distance between A and B is in [3.5, 4.5];*

  *A* = *B.*

(2) Gaps in the middle

*A* = *the last covered residue before gap;*

*B* = *the first covered residue after gap;*

*While there is gap*

*  Calculate the distance between A and B as d*_*AB;*_

*  Sample points around A;*

  Find point C where there is no or least atom clash, the distance between A and C is in [3.5, 4.5], and the distance between B and C is in [max(3.5, dd*(k-1)), max(4.5, max(3.8*k, dd*k))]. Here k is the number of remaining gaps and dd is d_*AB*_/*(k* + *1);*

  *A* = *C.*

Figure 12d demonstrates how the two kinds of gaps are filled.

### Packing other main chain atoms and side chain atoms

The model constructed for the target protein using the method described above only contains the coordinates of Cα atoms (i.e. Cα trace). Other main chain atoms (C, N, O) and side chain atoms need to be added. We use Pulchra^49^ to add other main chain atoms (C, N, O) and SCWRL4.0^50^ to add side chain atoms according to residue types.

## Additional Information

**How to cite this article**: Li, J. and Cheng, J. A Stochastic Point Cloud Sampling Method for Multi-Template Protein Comparative Modeling. *Sci. Rep.* **6**, 25687; doi: 10.1038/srep25687 (2016).

## Figures and Tables

**Table 1:** The average GDT-TS score, TM-score, GDT-HA score, and RMSD of the models predicted by MTMG using the first single templates and multiple templates on CASP9, CASP10, and CASP11 targets.

Dataset	Template	GDT-TS	TM	GDT-HA	RMSD
CASP11	Single	0.3906	0.4468	0.2739	16.16
	Multiple	0.4155	0.4700	0.2951	14.80
CASP10	Single	0.5366	0.5749	0.4111	11.09
	Multiple	0.5562	0.5930	0.4314	9.90
CASP9	Single	0.5370	0.5827	0.3851	10.61
	Multiple	0.5529	0.5968	0.3985	9.83

**Table 2:** The p-values of t-test on GDT-TS score and TM-score for the comparisons between using first single templates and using multiple templates on CASP9, CASP10, and CASP11 targets.

Dataset	p-value of GDT-TS score	p-value of TM-score
CASP11	2.355e-13	3.356e-12
CASP10	2.423e-07	1.087e-06
CASP9	2.227e-08	2.745e-07

**Table 3:** The average GDT-TS score, TM-score, GDT-HA score, and RMSD of the models predicted by MTMG and Modeller on CASP9, CASP10, and CASP11 targets.

Dataset	Method	GDT-TS	TM	GDT-HA	RMSD
CASP11	Modeller	0.4150	0.4641	0.2967	20.76
	MTMG	0.4155	0.4700	0.2951	14.80
CASP10	Modeller	0.5584	0.5889	0.4361	23.19
	MTMG	0.5562	0.5930	0.4314	9.90
CASP9	Modeller	0.5569	0.5992	0.4034	13.74
	MTMG	0.5529	0.5968	0.3985	9.83

**Table 4:** The average GDT-TS score, TM-score, GDT-HA score, and RMSD of the models predicted by MTMG and the probabilistic multi-template protein homology modeling method based on HHSearch sequence alignments on CASP9, 10 and 11 targets.

Dataset	Method	GDT-TS	TM	GDT-HA	RMSD
CASP11	MTMG	0.4256	0.4801	0.3010	11.62
	the probabilistic multi-template method	0.4322	0.4797	0.3112	14.42
CASP10	MTMG	0.5478	0.5897	0.4146	9.57
	the probabilistic multi-template method	0.5604	0.5957	0.4281	13.96
CASP9	MTMG	0.5536	0.5953	0.3970	9.20
	the probabilistic multi-template method	0.5669	0.6062	0.4128	12.45

**Table 5:** The average GDT-TS score of the models predicted by MTMG and Modeller on four protein categories on CASP9, 10, and 11 targets.

Dataset	Category	Modeller	MTMG
CASP11	all-alpha	0.3114	0.3195
	all-beta	0.4388	0.4429
	alpha/beta	0.6532	0.6392
	alpha+beta	0.3221	0.3255
CASP10	all-alpha	0.5907	0.5943
	all-beta	0.4526	0.4542
	alpha/beta	0.6670	0.6565
	alpha+beta	0.5894	0.5836
CASP9	all-alpha	0.5109	0.5152
	all-beta	0.4522	0.4496
	alpha/beta	0.6884	0.6777
	alpha+beta	0.5992	0.5923

**Table 6:** The average GDT-TS score and RMSD of the models predicted by MTMG and Modeller for different kinds of CASP targets.

Dataset	Classification	GDT-TS		RMSD	
		Modeller	MTMG	Modeller	MTMG
CASP11	TBM	0.5418	0.5385	11.04	9.76
	TBM-hard	0.1921	0.1977	35.56	23.6
	FM	0.1476	0.1578	41.23	24.81
CASP10	TBM	0.5824	0.5792	11.67	9.24
	FM/TBM	0.3181	0.3186	40.84	14.57
	FM	0.3099	0.3195	47.52	17.36
CASP9	TBM	0.6382	0.6338	8.85	6.86
	FM/TBM	0.1850	0.1865	27.29	21.37
	FM	0.1844	0.1861	36.47	23.53

## References

[b1] Eisenhaber F., Persson B. & Argos P. Protein structure prediction: recognition of primary, secondary, and tertiary structural features from amino acid sequence. Crit. Rev. Biochem. Mol. Biol. 30, 1–94 (1995). 7587278 10.3109/10409239509085139

[b2] Rost B. Protein structure prediction in 1D, 2D, and 3D. The Encyclopaedia of Computational Chemistry 3, 2242–2255 (1998).

[b3] Floudas C. Computational methods in protein structure prediction. Biotechnol. Bioeng. 97, 207–213 (2007). 17455371 10.1002/bit.21411

[b4] Lundström J., Rychlewski L., Bujnicki J. & Elofsson A. Pcons: a neural-network-based consensus predictor that improves fold recognition. Protein Sci. 10, 2354–2362 (2001). 11604541 10.1110/ps.08501 PMC2374055

[b5] Wallner B., Fang H. & Elofsson A. Automatic consensus-based fold recognition using Pcons, ProQ, and Pmodeller. Proteins: Struct. Funct. Bioinform. 53, 534–541 (2003). 10.1002/prot.10536 14579343

[b6] Källberg M. *et al.* Template-based protein structure modeling using the RaptorX web server. Nat. Protoc. 7, 1511–1522 (2012). 22814390 10.1038/nprot.2012.085 PMC4730388

[b7] McGuffin L. J. The ModFOLD server for the quality assessment of protein structural models. Bioinformatics 24, 586–587 (2008). 18184684 10.1093/bioinformatics/btn014

[b8] Zhou H. & Zhou Y. Fold recognition by combining sequence profiles derived from evolution and from depth‐dependent structural alignment of fragments. Proteins: Struct. Funct. Bioinform. 58, 321–328 (2005). 10.1002/prot.20308 PMC1408319 15523666

[b9] Jones D. GenTHREADER: an efficient and reliable protein fold recognition method for genomic sequences. J. Mol. Biol. 287, 797–815 (1999). 10191147 10.1006/jmbi.1999.2583

[b10] Roy A., Kucukural A. & Zhang Y. I-TASSER: a unified platform for automated protein structure and function prediction. Nat. Protoc. 5, 725–738 (2010). 20360767 10.1038/nprot.2010.5 PMC2849174

[b11] Li J. *et al.* The MULTICOM protein tertiary structure prediction system. Methods Mol. Biol. 1137, 29–41 (2014). 24573472 10.1007/978-1-4939-0366-5_3

[b12] Peng J. & Xu J. A multiple-template approach to protein threading. Proteins: Struct. Funct. Bioinform. 79, 1930–1939 (2011). 10.1002/prot.23016 PMC3092796 21465564

[b13] Meier A. & Söding J. Automatic Prediction of Protein 3D Structures by Probabilistic Multi-template Homology Modeling. Plos Comp. Biol. 11, e1004343 (2015). 10.1371/journal.pcbi.1004343 PMC4619893 26496371

[b14] Baú D. *et al.* Distill: a suite of web servers for the prediction of one-, two-and three-dimensional structural features of proteins. BMC Bioinformatics 7, 402 (2006). 16953874 10.1186/1471-2105-7-402 PMC1574355

[b15] Simons K., Kooperberg C., Huang E. & Baker D. Assembly of protein tertiary structures from fragments with similar local sequences using simulated annealing and Bayesian scoring functions. J. Mol. Biol. 268, 209–225 (1997). 9149153 10.1006/jmbi.1997.0959

[b16] Sánchez R. & Sali A. Evaluation of comparative protein structure modeling by MODELLER-3. Proteins: Struct. Funct. Genet. 29, 50–58 (1997). 9485495 10.1002/(sici)1097-0134(1997)1+<50::aid-prot8>3.3.co;2-w

[b17] Venclovas Č. & Margelevičius M. Comparative modeling in CASP6 using consensus approach to template selection, sequence-structure alignment, and structure assessment. Proteins: Struct. Funct. Bioinform. 61, 99–105 (2005). 10.1002/prot.20725 16187350

[b18] Larsson P., Wallner B., Lindahl E. & Elofsson A. Using multiple templates to improve quality of homology models in automated homology modeling. Protein Sci. 17, 990–1002 (2008). 18441233 10.1110/ps.073344908 PMC2386743

[b19] Šali A. & Blundell T. L. Comparative protein modelling by satisfaction of spatial restraints. J. Mol. Biol. 234, 779–815 (1993). 8254673 10.1006/jmbi.1993.1626

[b20] Fiser A. & Sali A. Modeller: generation and refinement of homology-based protein structure models. Methods Enzymol. 374, 461–491 (2003). 14696385 10.1016/S0076-6879(03)74020-8

[b21] Schwede T., Kopp J., Guex N. & Peitsch M. SWISS-MODEL: an automated protein homology-modeling server. Nucleic Acids Res. 31, 3381 (2003). 12824332 10.1093/nar/gkg520 PMC168927

[b22] Levitt M. Accurate modeling of protein conformation by automatic segment matching. J. Mol. Biol. 226, 507–533 (1992). 1640463 10.1016/0022-2836(92)90964-l

[b23] Petrey D. *et al.* Using multiple structure alignments, fast model building, and energetic analysis in fold recognition and homology modeling. Proteins: Struct. Funct. Bioinform. 53, 430–435 (2003). 10.1002/prot.10550 14579332

[b24] Cheng J. A multi-template combination algorithm for protein comparative modeling. BMC Struct. Biol. 8, 18 (2008). 18366648 10.1186/1472-6807-8-18 PMC2311309

[b25] Zhang J. & Zhang Y. A novel side-chain orientation dependent potential derived from random-walk reference state for protein fold selection and structure prediction. Plos One 5, e15386 (2010). 21060880 10.1371/journal.pone.0015386 PMC2965178

[b26] Zemla A. LGA: a method for finding 3D similarities in protein structures. Nucleic Acids Res. 31, 3370–3374 (2003). 12824330 10.1093/nar/gkg571 PMC168977

[b27] Zhang Y. & Skolnick J. Scoring function for automated assessment of protein structure template quality. Proteins: Struct. Funct. Bioinform. 57, 702–710 (2004). 10.1002/prot.20264 15476259

[b28] Cozzetto D. *et al.* Evaluation of template-based models in CASP8 with standard measures. Proteins: Struct. Funct. Bioinform. 77, 18–28 (2009). 10.1002/prot.22561 PMC4589151 19731382

[b29] Huang Y. J., Mao B., Aramini J. M. & Montelione G. T. Assessment of template-based protein structure predictions in CASP10. Proteins: Struct. Funct. Bioinform. 82, 43–56 (2014). 10.1002/prot.24488 PMC3932189 24323734

[b30] Li J., Deng X., Eickholt J. & Cheng J. Designing and benchmarking the MULTICOM protein structure prediction system. BMC Struct. Biol. 13, 2 (2013). 23442819 10.1186/1472-6807-13-2 PMC3599124

[b31] Cheng J., Li J., Wang Z., Eickholt J. & Deng X. The MULTICOM toolbox for protein structure prediction. BMC Bioinformatics 13, 65 (2012). 22545707 10.1186/1471-2105-13-65 PMC3495398

[b32] Wang Z., Eickholt J. & Cheng J. MULTICOM: a multi-level combination approach to protein structure prediction and its assessments in CASP8. Bioinformatics 26, 882–888 (2010). 20150411 10.1093/bioinformatics/btq058 PMC2844995

[b33] Li J., Adhikari B. & Cheng J. An improved integration of template-based and template-free protein structure modeling methods and its assessment in CASP11. Protein Pept. Lett. 22, 586–593 (2015). 25990081 10.2174/0929866522666150520145717 PMC4593487

[b34] Cao R., Bhattacharya D., Adhikari B., Li J. & Cheng J. Large-scale model quality assessment for improving protein tertiary structure prediction. Bioinformatics 31, i116–i123 (2015). 26072473 10.1093/bioinformatics/btv235 PMC4553833

[b35] Li J., Cao R. & Cheng J. A large-scale conformation sampling and evaluation server for protein tertiary structure prediction and its assessment in CASP11. BMC Bioinformatics 16, 337 (2015). 26493701 10.1186/s12859-015-0775-x PMC4619059

[b36] Soding J. Protein homology detection by HMM-HMM comparison. Bioinformatics 21, 951–960 (2005). 15531603 10.1093/bioinformatics/bti125

[b37] Finn R. D., Clements J. & Eddy S. R. HMMER web server: interactive sequence similarity searching. Nucleic Acids Res. 39, W29–W37 (2011). 21593126 10.1093/nar/gkr367 PMC3125773

[b38] Biegert A. & Söding J. Sequence context-specific profiles for homology searching. Proc. Natl. Acad. Sci. USA. 106, 3770–3775 (2009). 19234132 10.1073/pnas.0810767106 PMC2645910

[b39] Welch B. L. The generalization of “student’s” problem when several different population variances are involved. Biometrika 34, 28–35 (1947). 20287819 10.1093/biomet/34.1-2.28

[b40] Chen V. B. *et al.* MolProbity: all-atom structure validation for macromolecular crystallography. Acta Crystallogr. D66, 12–21 (2010). 10.1107/S0907444909042073 PMC2803126 20057044

[b41] Xu D. & Zhang Y. Improving the physical realism and structural accuracy of protein models by a two-step atomic-level energy minimization. Biophys. J. 101, 2525–2534 (2011). 22098752 10.1016/j.bpj.2011.10.024 PMC3218324

[b42] Tong Y. L. The Multivariate Normal Distribution. 23–61 (Springer, 1990).

[b43] Gentle J. E. Computational statistics. 315–316 (Springer, 2009).

[b44] Venables W. N. & Ripley B. D. Modern applied statistics with S. (Springer, 2002).

[b45] Kirkpatrick S., Gelatt C. D. Jr & Vecchi M. P. Optimization by Simulated Annealing. Science 220, 671–680 (1983). 17813860 10.1126/science.220.4598.671

[b46] Černý V. Thermodynamical approach to the Traveling Salesman Problem: an efficient simulation algorithm. J. Optim. Theory Appl. 45, 41–51 (1985).

[b47] Taylor C. J. & Kriegman D. J. Minimization on the Lie Group SO(3) and related manifolds. No. 9405 (Technical Report, 1994).

[b48] Henikoff S. & Henikoff J. Amino acid substitution matrices from protein blocks. *Proc. Natl. Acad. Sci.* USA. 89, 10915–10919 (1992). 1438297 10.1073/pnas.89.22.10915 PMC50453

[b49] Rotkiewicz P. & Skolnick J. Fast procedure for reconstruction of full-atom protein models from reduced representations. J. Comput. Chem. 29, 1460–1465 (2008). 18196502 10.1002/jcc.20906 PMC2692024

[b50] Krivov G. G., Shapovalov M. V. & Dunbrack R. L. Improved prediction of protein side-chain conformations with SCWRL4. Proteins: Struct. Funct. Bioinform. 77, 778–795 (2009). 10.1002/prot.22488 PMC2885146 19603484

